# Transformation of chronic disease management: Before and after the COVID-19 outbreak

**DOI:** 10.3389/fpubh.2023.1074364

**Published:** 2023-03-29

**Authors:** Steven Yu, Rongjun Wan, Lu Bai, Bingrong Zhao, Qiaoling Jiang, Juan Jiang, Yuanyuan Li

**Affiliations:** ^1^Department of Respiratory Medicine, National Key Clinical Specialty, Branch of National Clinical Research Center for Respiratory Disease, Xiangya Hospital, Central South University, Changsha, China; ^2^Center of Respiratory Medicine, Xiangya Hospital, Central South University, Changsha, China; ^3^Clinical Research Center for Respiratory Diseases in Hunan Province, Changsha, China; ^4^Hunan Engineering Research Center for Intelligent Diagnosis and Treatment of Respiratory Disease, Changsha, China; ^5^National Clinical Research Center for Geriatric Disorders, Xiangya Hospital, Changsha, China

**Keywords:** COVID-19, chronic disease management, mobile health, telemedicine, technology-based education

## Abstract

Adults with chronic diseases often experience a decline in their quality of life along with frequent exacerbations. These diseases can cause anxiety and impose a significant economic burden. Self-management is a crucial aspect of treatment outside of the hospital and can improve quality of life and reduce the financial burden resulting from unexpected hospitalizations. With the COVID-19 pandemic, telehealth has become a vital tool for both medical professionals and patients; many in-person appointments have been canceled due to the pandemic, leading to increased reliance on online resources. This article aimed to discuss various methods of chronic disease management, both traditional self-management and modern telehealth strategies, comparing before and after the COVID-19 outbreak and highlighting challenges that have emerged.

## 1. Introduction

Chronic diseases such as hypertension, diabetes, and chronic obstructive pulmonary disease are major causes of disability worldwide. Almost one in three adults suffer from at least one chronic condition, and research has suggested that 16–57% of adults in developed countries suffer from multiple chronic conditions (1). Disease management, such as persistent monitoring of vital signs, screening of biomarkers, and lifestyle modification can reduce premature mortality from chronic diseases (2). Chronic disease management often requires a patient-professional partnership, including self-management education, which traditionally provides only information and technical skills; however, current self-management education offers problem-solving skills that emphasize the enhancement of self-efficacy, improved health outcomes, and reduced costs for patients (3).

**Figure 1:**
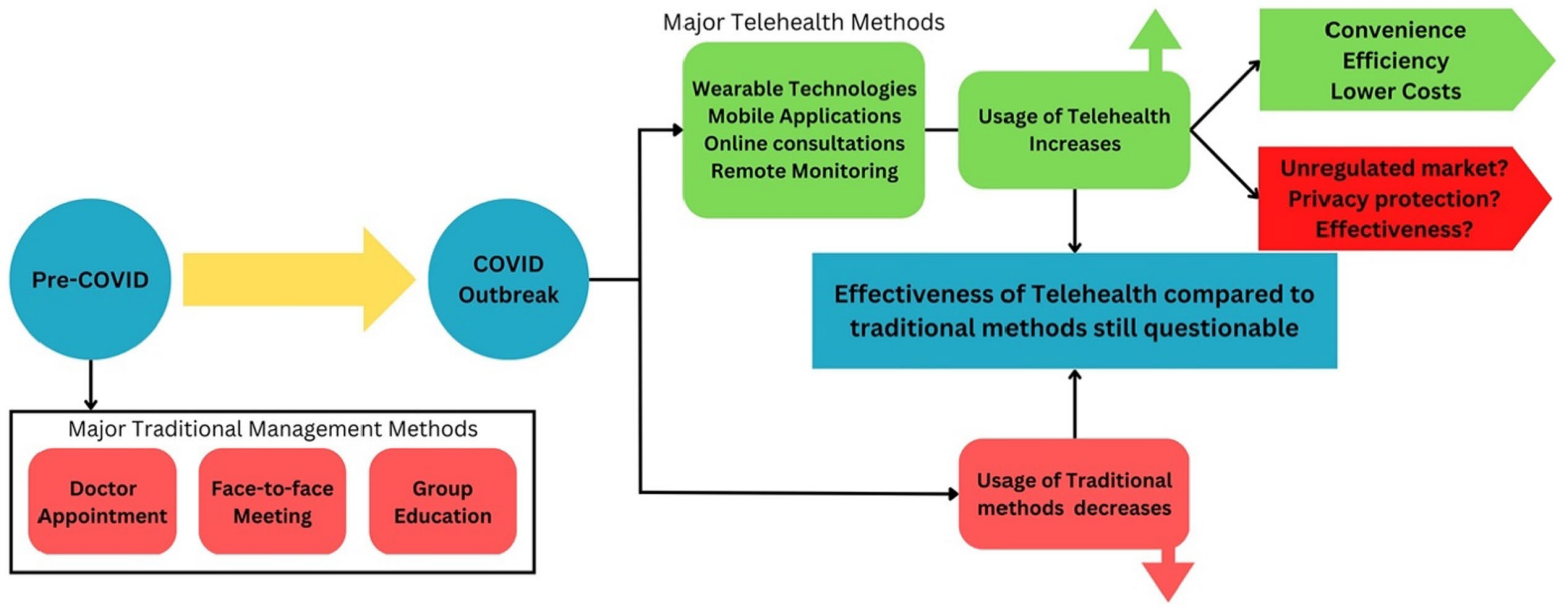
The shift of chronic disease management method after COVID-19 outbreak. Following the outbreak of COVID-19, there was a significant increase in the use of telehealth, which brought convenience and benefits to patients, but also raised several concerns, such as an unregulated market, privacy protection, and the effectiveness of telehealth. Simultaneously, the use of traditional chronic disease management methods has decreased significantly. Whether or not telehealth can truly replace traditional methods remains to be determined, in addition to the effectiveness of each approach.

After the global outbreak of the COVID-19 pandemic, patients with chronic diseases had difficulty attending outpatient meetings and, in some cases, needed to postpone or cancel follow-ups. In response, outpatient visits and meetings for patients have mainly been moved to telehealth platforms due to personal preferences and potential contagion risk (4). The COVID-19 pandemic and the need for social distancing have further accelerated the rapid shift to technology-enabled patient education and healthcare interactions. Despite the convenience and reduced cost of healthcare in the pandemic era, whether the use of telemedicine can be a complete substitute for face-to-face meetings is still questionable (Figure 1).

## 2. The burden of chronic diseases

### 2.1. Patients

Individuals with chronic diseases face physical and psychological inconveniences due to persistent symptoms. Chronic diseases have a major impact on patients’ health-related quality of life and are associated with decreased function, increased mortality risk, and higher costs of personal medical care (5, 6). For individuals with low socioeconomic status, chronic diseases can be perceived as an extra burden and distraction in addition to poverty, social isolation, and poor education (7). Patients with chronic diseases often exhibit psychological symptoms, such as anxiety and depression, which have adverse effects on their conditions (8). Furthermore, interruptions in care and other challenges related to the COVID-19 pandemic may lead to poorer mental health outcomes in patients with chronic diseases (9).

### 2.2. Society

Chronic diseases impose a tremendous burden on society and the economy. It is estimated that by 2030, the total cost of chronic diseases in the United States will cumulatively exceed 42 trillion USD, with an additional 794 billion USD per year of losses resulting from the loss of employee productivity (10). Chronic diseases are responsible for the death of approximately 41 million people each year, accounting for 74% of global deaths (11). Several studies have also indicated that mortality rates of potentially preventable diseases (including chronic diseases) are higher in low- and middle-income countries than in those with high income (12, 13).

## 3. Traditional disease-specific chronic disease self-management intervention

### 3.1. Diabetes mellitus

Diabetes is a chronic disease that requires intervention, self-management, education, and support for patients to improve their daily quality of life and outcomes (14). According to the American Association of Diabetes Education, diabetes self-care can be divided into seven categories: healthy eating, being active, health monitoring, taking medication, problem-solving, healthy coping, and reducing risks (15). A study in the US showed that medical costs are about 2.3 times higher for patients who are diagnosed compared to those who are not (16). A randomized controlled trial conducted by Eroglu et al. (17) designed to evaluate the effect of diabetes self-management education among patients with type II diabetes reported that after a 6-month follow-up with an educational program, individuals in the intervention group had more controlled diabetes and higher scores for self-efficacy. The diabetes community has been and remains heavily impacted by the COVID-19 pandemic; a meta-analysis in China found that 9.7% of COVID-19 patients with coexisting diabetes had triple the risk of developing severe disease (18).

### 3.2. Chronic obstructive pulmonary disease

Chronic obstructive pulmonary disease (COPD) is a prevailing threat in modern society, mostly causing chronic cough, dyspnea, and exhaustion. It is ranked as the third leading cause of death worldwide and caused 3.23 million deaths in 2019 (19). A systematic review by Effing et al. summarized that COPD self-management programs can be categorized as either smoking cessation support, self-recognition and treatment of exacerbations, increased exercise, nutritional advice, or dyspnea management (20). For long-term impact and sustainability, education provided by professionals to patients, health care workers, and families is best to ensure enforcement and efficiency (21). Most education requires patients to attend face-to-face meetings and receive interventions. One beneficial therapeutic approach that can be used to assist in improving psychological and physical outcomes is cognitive-behavioral therapy (CBT), where patients work with therapists to exchange their thoughts and understanding of symptoms, mentality, and knowledge about the disease (20). Compared with usual care for COPD, intensive CBT has shown greater improvement in psychological and physical symptoms (22); however, cessation of non-pharmacological interventions, including rehabilitation, has become a dilemma for patients during the pandemic. Challenges that have emerged are whether or not the traditional algorithms of pharmacological management in COPD still work, as well as how pandemic-related limitations in non-pharmaceutical interventions can be overcome, as COVID-19 circulation increases healthcare utilization risk for these patients (23).

### 3.3. Cardiovascular diseases

Cardiovascular diseases (CVD) were the most common causes of death worldwide and led to almost 17.9 million deaths per year globally (24). The American Heart Association suggests that people undergo lifestyle changes to manage and prevent CVDs based on seven factors (Life’s Simple 7): smoking status, physical activity, body weight, diet, blood glucose, cholesterol, and blood pressure (25). It is essential for potential and diagnosed CVD patients to receive self-management interventions, as the diseases could be present but remain asymptomatic for years. The most common self-management for CVD patients is introduced in segments of self-responsibility, health status recognition, diet, weight control, aerobic exercise, smoking cessation, alcohol consumption, and medication adherence (26). During the pandemic, diet management has faced problems in decreases in food security and nutrition provision, which are detrimental to vulnerable patients with CVD (27).

### 3.4. Cancer

Traditional cancer self-management programs are mainly derived from focus groups and adapted from other chronic disease management programs (28). Research on the use of self-management interventions in cancer care has grown tremendously over the past few decades, resulting in significant changes in symptom management assistance during treatment to address both physical symptoms and psychosocial distress (29). The PROSELF Pain Control Program (PSPC), a psychoeducational self-management program that aims to assist patients in managing cancer-related pain, promotes self-assessment of pain, appropriate use of analgesics, and self-dosing to prevent pain escalation, providing participants with improved management of pain and a lower pain intensity score (30).

Even though the specific vulnerability of cancer patients to COVID-19 has yet to be determined (31), cancer patients are still recommended to receive stronger protection and more intensive surveillance than the normal population (32).

## 4. Acceleration of telehealth development during the COVID-19 outbreak

### 4.1. Telehealth encouraged as face-to-face visits decrease

At the beginning of the pandemic, strict and inconsistent quarantine policies caused patients with chronic diseases to suffer from changed lifestyles and medical routines. A survey conducted in the United States indicated that more than half of patients with chronic conditions felt that their lifestyles and routines, including medical plans, had been altered significantly (33). In Belo Horizonte, a major city in Brazil, the hospitalization rate for patients with cardiovascular diseases decreased by 16.3% from March to December 2020 in contrast to expectations (34). Similarly, 17.7% of patients with chronic diseases who participated in a survey in Japan canceled their face-to-face visits and experienced a shortage of drugs in April and May of 2020 (35).

The enthusiasm for telehealth adoption from patients with chronic diseases has increased during the pandemic. Patients, especially older adults, are now willing to pay the same amount of money for online remote sessions as face-to-face sessions (36). McKinsey and Company conducted a study that indicated increased use of telemedicine in recent periods; telehealth usage rates jumped from 11% of United States consumer users in 2019 to 46% in May 2020. In April 2020, the movement from office visits and outpatient care to telehealth skyrocketed 78 times higher than that seen in February. Data also show that stabilization of telehealth utilization increased 38 times compared to before the pandemic (37). A cross-sectional study conducted at the Royal College of General Practitioners Research Surveillance Centre found that face-to-face consultations fell by 64.6% and home visits fell by 62.6%, with almost a two-fold increase in telephone/electronic usage compared to the same timeframe during the early pandemic stages (38).

The COVID-19 pandemic has shifted government and public focus towards the use of digital and mobile apps (mAPPs) due to surging case numbers and strict quarantine measures. The global usage of mAPPs for health monitoring, education, and COVID-19 detection has skyrocketed, especially in densely populated countries in East and Southeast Asia (39). Mobile applications have matured and become increasingly used in chronic disease management for patient education, monitoring, and interactions for the last few decades (40), potentially showing a positive trend in patient adherence to chronic disease management (41). mAPPs have also been proven to be effective tools for reducing hospital burden, obtaining reliable information, tracking symptoms, and improving mental health (42).

Apart from traditional video conferences and phone calls to communicate with providers, telemonitoring combined with plentiful modern technologies has attracted more attention than usual during the pandemic (43). Wearable technology that can monitor physical activity, blood pressure, and other information has gained traction, allowing real-time synchronous and asynchronous data and biodata to be delivered to healthcare providers to provide more personalized and precise decisions for patients with chronic diseases (44, 45).

### 4.2. Telehealth usage for specific diseases

To continuously monitor clinical conditions, a special garment with an integrated sound acquisition module which directly decreased the patient’s role in operating the machine was implemented during the pandemic to capture thoracic sounds and monitor patients with COPD (46). To ensure the safety of sleep apnea patients and reduce the workload for medical workers in the COVID-19 era, a portable sleep apnea monitoring system that integrated with the mobile phones of doctors and patients’ relatives were designed, and patients were satisfied with the convenience, accuracy, and lower cost of the system (47). When technology-based pulmonary rehabilitation education was delivered to patients with chronic respiratory disease, no significant differences in quality of life and exercise ability were found compared to those who received traditional pulmonary rehabilitation, indicating that telehealth could be a trustworthy replacement during the pandemic era (48).

For diabetes, technology such as remote continuous glucose monitoring helped healthcare workers remotely grasp glucose level data for diabetic patients in both inpatient and outpatient wards. It has been proven to help the management of glycemic index levels during pandemics (49, 50). Healthcare workers and companies also turned to technology at the start of the pandemic, and diabetes-related educational applications and digital support groups including online courses and discussions were set up to help with patient care (51).

Known as Tele-CR, the delivery of cardiac rehabilitation online to help with goal setting, delivering self-management advice, and counseling for CVD patients was recommended by healthcare workers during the pandemic (52). Along with wearable trackers and telehealth tools, CR exercises could be conducted and prescribed remotely, leading to potential resource and cost savings for the healthcare system (53). Telehealth enhances the monitoring, tracking, and communication of biometric information, allowing hypertensive patients to participate better in their care which reduces their stress. Services can be easily used to notify referring physicians of the onset of acute symptoms or sudden increases in blood pressure (54).

Studies have suggested that telemedicine in cancer patient care leveraged innovative responses during the COVID-19 pandemic that may provide durable solutions that allow patients to receive proper care in their homes (55). In an randomized controlled trial conducted by Maguire et al., Advanced Symptom Management System, a remote monitoring system, was shown to be a highly efficient tool for improving the quality of life of individuals undergoing chemotherapy for various cancers. ASyMS enables real-time 24/7 monitoring and management of chemotherapy toxicity by collecting patient data and transmitting it to clinicians for evaluation. It has been demonstrated to have a positive impact on symptom burden, anxiety, self-efficacy, and other critical outcomes in patients undergoing chemotherapy; additionally, it supports patients who remain at home to receive optimal care by providing a secure and reliable platform during health crises (56).

### 4.3. Telehealth and COVID-19

Long-COVID is a newly emerging phenomenon that refers to persistent physical and neuropsychiatric symptoms after COVID-19 infection that last over 12 weeks without a clear cause (57), causing a health burden of up to 30% across all age groups and can potentially impact the healthcare system and economy (58). A 10-week virtual rehabilitation program for Long-COVID patients, led by multidisciplinary team, conducted weekly one-hour video learning sessions and peer interaction on Long-COVID symptoms and education. Participants have highly regarded the program, especially the guidance on breathlessness and fatigue management (59). Furthermore, a project that incorporated standardizing patient assessments, ensuring individual rehabilitation plans, and reporting activity performance was conducted in Italy, showing improvement for Long-COVID patient and caregiver education and creation of a regional database for data collection (60). Additionally, the use of telehealth could effectively assess patients with Long-COVID, providing a convenient and reliable means of remote health assessment in the absence of established guidelines or diagnostic procedures. Advanced analysis implemented in telehealth could detect disorders and facilitate further diagnostic evaluation, thereby also reducing the stress associated with Long-COVID (61).

Inadequate telehealth training among healthcare workers is another concern (62). Especially in the current phase of COVID-19 when quarantine and lockdown are no longer recommended, it is necessary to increase the number of trained and educated telehealth workers and provide them with rigorous training (63). Moreover, Covid-19 will not be the first or the last major infectious disease or natural disaster. A recent study suggests several ways to improve the utilization of telehealth during similar situations in the present or future, including training healthcare professionals, introducing accreditation and funding services, redesigning care models, and integrating telehealth into routine clinical work (64). Determining a path for utilizing telehealth after the pandemic can motivate and guide researchers, medical and government personnel to make advanced contributions on the telehealth area (65). A study proposed that telehealth should be widely and permanently implemented to achieve significant public health benefits such as reducing workload for physicians and alleviating patient flow during the current pandemic and in the future (66). However, given that both COVID-19 infection and its post-acute syndrome are relatively new phenomena, additional research and optimization of their relationship with telehealth are necessary.

### 4.4. Emerging problems

As telehealth is still in its youngest stage, patient satisfaction depends on the modality and functionality of delivery; however, current conclusions surrounding telehealth platform efficiency and effectiveness are uncertain (67). The effectiveness of telehealth in individuals with complicated and mixed chronic disease conditions remains unclear, thus self-management and clinical decision-making are not recommended as the main components of telehealth for patients with complex chronic conditions (68). Even though telehealth could maintain the doctor-patient relationship during the pandemic, patients still suggested that a combination of telehealth and offline face-to-face meetings would be preferable in the future (69).

Notably, telehealth platforms pose ethical and legal issues than traditional management, surrounding the liability of professionals, quality of care, and protection of personal data (70). Even in developed countries, policies and regulations associated with telehealth privacy are underregulated. The Health Insurance Portability and Accountability Act, a United States federal law enacted almost two decades ago that protects patients’ health privacy, has barely been updated since it came into law (71). Furthermore, the quality of mobile applications with diagnosing and rating functions has been found to be spotty, functionally inaccurate, and inefficient. Frequent updates and adjustments to the mAPP from the operator could pose a further barrier for government regulators to supervise the mAPP update in a timely manner (72). In addition to regulations on privacy and safety, informed consent is another essential component of telehealth; patients not only have to understand their rights and have them fulfilled, but providers must also understand the purpose of their actions, acquire consent from consumers, and ensure that patients understand every instruction (73). Access to telemedicine also poses challenges to users. A recent study indicated that females and families with relatively lower household incomes tended to participate less in telemedicine for CVD during the COVID-19 pandemic (74). Another less-active group was older adults, the majority of whom could not use video visits and other telemedicine methods due to lower levels of education, living alone, and low electronic literacy (75). Seniors’ experiences with telehealth are still evolving, and innovative technologies that address their needs must be explored to increase telehealth usage and acceptance (76).

With the existing unsolved barriers in this fast-developing field, ethical and legal issues still require more standardization and regulation to guarantee patients’ rights and quality of care. Groups that are often overlooked should also be a focus of attention for healthcare workers and telehealth companies.

## 5. Discussion

This review discussed and analyzed modern chronic disease management strategies before and after the COVID-19 pandemic. The shift from traditional in-person care to technology-based telehealth management has been obvious, as an increasing number of patients have opted for telehealth over traditional clinical visits. It is expected that if more novel communication channels between patients and doctors can be developed, communication will become smoother, easier, and more efficient. Cutting-edge technology and telehealth have resulted in improved patient adherence to programs and convenience for users, but despite its demonstrated benefits, uncertainties regarding the quality of privacy protection and safety remain. The overall impact of telehealth during the pandemic has yet to be determined, and patients and healthcare workers must collaborate to find the best solutions for disease management. This article intends to contribute to the advancement and evolution of chronic disease management methods. While the pandemic is expected to end at some point, the accomplishments made in the field of telehealth during this period must not be neglected and should be built upon to further refine chronic disease management approaches.

## Author contributions

SY: conceptualization, writing, revision, and approval of the final version. JJ and YL: revision and approval of the final version. RW: revision and writing. LB, QJ, and BZ: revision and methodology. All authors have contributed to the manuscript and approved the submitted version.

## Funding

This work was supported by grants from the Respiratory and Critical Care Medicine Department, National Key Clinical Specialty Construction Project (Z047-02), National Natural Science Foundation of China (82100099 and 82170041), and Innovative Research Platform of Hunan Development and Reform Commission (2021-212).

## Conflict of interest

The authors declare that the research was conducted in the absence of any commercial or financial relationships that could be construed as a potential conflict of interest.

## Publisher’s note

All claims expressed in this article are solely those of the authors and do not necessarily represent those of their affiliated organizations, or those of the publisher, the editors and the reviewers. Any product that may be evaluated in this article, or claim that may be made by its manufacturer, is not guaranteed or endorsed by the publisher.
